# Akt Mediates Metastasis-Associated Gene 1 (MTA1) Regulating the Expression of E-cadherin and Promoting the Invasiveness of Prostate Cancer Cells

**DOI:** 10.1371/journal.pone.0046888

**Published:** 2012-12-05

**Authors:** Hongyan Wang, Liangsheng Fan, Juncheng Wei, Yanjie Weng, Li Zhou, Ying Shi, Wenjuan Zhou, Ding Ma, Changyu Wang

**Affiliations:** 1 Cancer Biology Research Center, Tongji Hospital, Huazhong University of Science and Technology, Wuhan, Hubei, PR China; 2 Department of Gynecologic Oncology, Tongji Hospital, Huazhong University of Science and Technology, Wuhan, Hubei, PR China; 3 Department of Obstetrics and Gynecology, Qilu Hospital, Shandong University, Jinan, Shandong, PR China; 4 Tianjin Central Hospital of Obstetrics and Gynecology, Tianjin, PR China; 5 Department of Obstetrics and Gynecology, The First Affiliated Hospital of Guangzhou Medical College, Guangzhou, PR China; Southern Illinois University School of Medicine, United States of America

## Abstract

Human metastasis-associated gene 1 (MTA1) is highly associated with the metastasis of prostate cancer; however, the molecular functions of MTA1 that facilitate metastasis remain unclear. In this study, we demonstrate that the silencing of MTA1 by siRNA treatment results in the upregulation of E-cadherin expression by the phosphorylation of AKT (p-AKT) and decreases the invasiveness of prostate cancer cells. We show that MTA1 is expressed in over 90% of prostate cancer tissues, especially metastatic prostate cancer tissue, comparing to non-expression in normal prostate tissue. RT-PCR analysis and Western blot assay showed that MTA1 expression is significantly higher in highly metastatic prostate cancer PC-3M-1E8 cells (1E8) than in poorly metastatic prostate cancer PC-3M-2B4 cells (2B4). Silencing MTA1 expression by siRNA treatment in 1E8 cells increased the cellular malignant characters, including the cellular adhesive ability, decreased the cellular invasive ability and changed the polarity of cellular cytoskeleton. 1E8 cells over-expressing MTA1 had a reduced expression of E-cadherin, while 1E8 cells treated with MTA1 siRNA had a higher expression of E-cadherin. The expression of phosphorylated AKT (p-AKT) or the inhibition of p-AKT by wortmannin treatment (100 nM) significantly altered the function of MTA1 in the regulation of E-cadherin expression. Alterations in E-cadherin expression changed the role of p-AKT in cellular malignant characters. All of these results demonstrate that MTA1 plays an important role in controlling the malignant transformation of prostate cancer cells through the p-AKT/E-cadherin pathway. This study also provides a new mechanistic role for MTA1 in the regulation of prostate cancer metastasis.

## Introduction

Prostate cancer is one of the most common malignant cancers and is the second leading cause of cancer deaths in American men [1]. Treatment failure occurs for prostate cancer often due to lymph node and/or distant organ metastasis. The molecular mechanisms of prostate cancer metastasis and invasion are not well elucidated. Increasing evidence has indicated that the human metastasis-associated gene 1 (MTA1) is a key factor in tumor metastasis [2]. One study that used a serological analysis of recombinant cDNA expression libraries (SEREX) found that MTA1 was preferentially expressed in a panel of malignant prostate carcinoma tissues compared with normal tissues, which suggests that MTA1 may be required for prostate carcinoma metastasis [3] Another study found that MTA1 was selectively over-expressed in metastatic prostate cancer compared to clinically localized prostate cancer and benign prostate tissue [4]. These studies demonstrated that MTA1 may play an important role in the metastasis of prostate cancer; however, the mechanistic role of MTA1 in the process of prostate cancer metastasis is still poorly understood.

MTA1 was originally identified by differential cDNA screening using highly metastatic breast cancer cell lines [5]. The MTA1 gene encodes a novel protein that contains a proline-rich region (SH3-binding motif), a putative zinc finger motif, a leucine zipper motif and 5 copies of the SPXX DNA-binding motif [6]. The MTA1 protein has been found in the nucleosome remodeling histone deacetylase (NuRD) complex, which has been shown to modify or remodel chromosomes [7]. MTA1 physically interacts with histone deacetylase (HDAC), which plays an important role in histone deacetylation and the alteration of transcriptional control [8]. As an important regulator of cell fate with a role in the oncogenesis and progression of many malignant tumors, MTA1 has attracted widespread attention [2].

**Figure 1:**
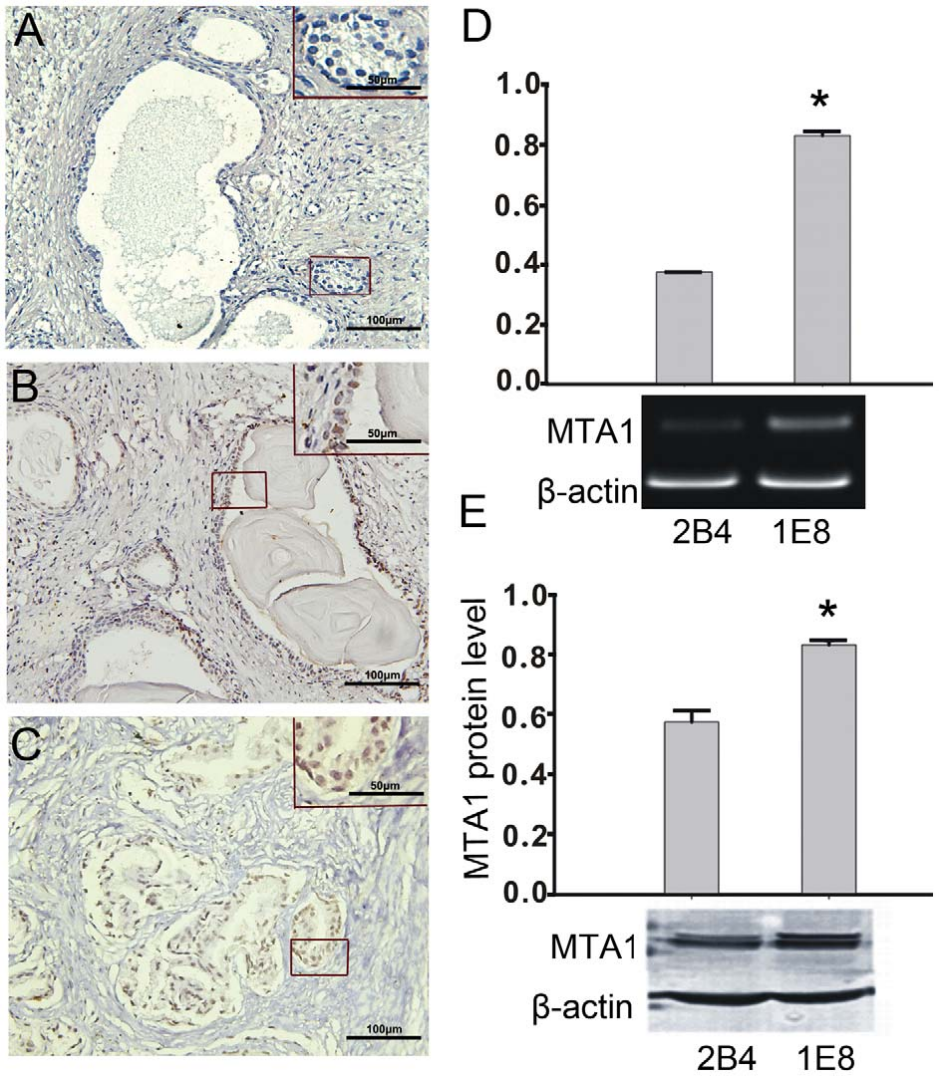
The expression of MTA1 in prostate cancer tissue and prostate cancer cells. (A–C) Typical results for the MTA1 staining pattern determined by immunohistochemistry are shown in normal prostate tissue (A), localized prostate cancer tissue (B), and metastatic prostate cancer tissue (C). The results in B and C showed MTA1 expression in both nuclei and cytoplasm. The original magnification for A–C is 200 fold and detailed with enlarged view is 400 fold. (D) A quantitative RT-PCR analysis of the MTA1 RNA levels in 2B4 and 1E8 cells (top). An asterisk (*) indicates a statistically significant difference (p<0.05) compared to the 2B4 cells. (E) The protein expression levels of MTA1 in 2B4 and 1E8 cells were analyzed by Western blotting using specific antibodies, and the results were quantified (top). An asterisk (*) indicates a statistically significant difference (p<0.05) compared to the 2B4 cells. The experiments were repeated three times.

For epithelial cells to develop into cancer cells, the epithelial mesenchymal transition (EMT) must occur [9]. The EMT leads epithelial cell layers to lose polarity and cell-cell contacts and triggers the remodeling of the cellular cytoskeleton [10], [11]. E-cadherin is regarded as a main indicator of the occurrence of the EMT [12]. E-cadherin plays important roles in malignant phenotypes, including cell adhesion, cellular differentiation, and cell structure. The upregulation of E-cadherin has been implicated in the inactivation of the EMT [13], [14]. Therefore, E-cadherin has been suggested to serve as a strong tumor suppressor in cancer development [14], [15]. Histone deacetylation and/or the hypermethylation of the CpG islands in E-cadherin have been shown to be the main mechanisms for E-cadherin silencing in tumors [15], [16], [17]. MTA1 has been shown to have a role in histone deacetylation, the alteration of chromatin structure and transcriptional control [18], [19]. These results suggest that MTA1 may possibly regulate E-cadherin. Moreover, MTA1 has been shown to influence EMT phenotypes [20], [21]. Previously, we have shown that E-cadherin expression is upregulated in melanoma and cervical cancer cells treated with MTA1 siRNA [22]. However, these studies did not focus on the mechanism regulating the changes in E-cadherin gene expression by MTA1. The phosphatidylinositol 3-kinase (PI3K)/AKT pathway is believed to play an important role in human cancer progression, including the progression of prostate cancer [23], [24]. MTA1 has been found to regulate AKT expression [25]. To change the malignant phenotype of cancer cells, the MTA1/AKT pathway may activate genes and antagonize genes that suppress proliferation and/or cell metastasis. Recently, the PI3K/AKT pathway has been shown to be a central regulator of the EMT [26], [27]. E-cadherin is also a key regulator of the EMT and an inhibitor of cancer development that can be regulated by the PI3K/AKT pathway [28]. In this study, we demonstrate that MTA1 changes the malignant phenotype of prostate cancer cells and regulates the expression of E-cadherin by an AKT phosphorylation-dependent mechanism. This preclinical study provides a better understanding of the roles of MTA1 and forms the basis for further clinical study of MTA1 as a target gene.

**Figure 2:**
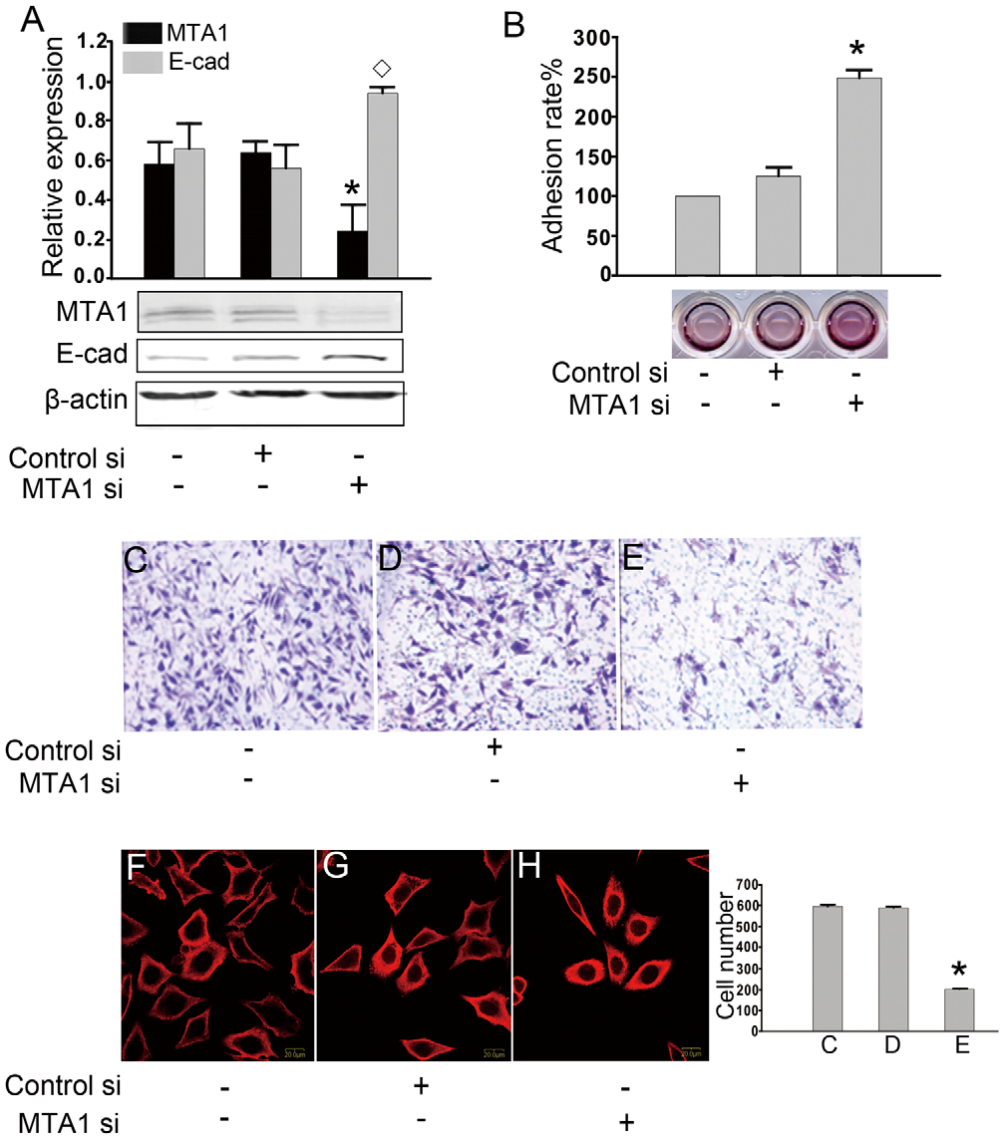
The influence of MTA1 expression on the malignant phenotypes of 1E8 cells. (A) Treatment with MTA1 siRNA decreased MTA1 expression efficiently and enhanced E-cadherin expression. The protein levels were analyzed using Western blotting and were normalized to β-actin. The quantification of the band intensities is shown (top). An asterisk (*) or diamond (◊) indicates a statistically significant difference (p<0.05) in the MTA1 or E-cadherin levels, respectively, compared with the nontreated cells and the negative-control siRNA-transfected cells, n = 3. (B) The adhesive rate was quantified by the MTT assay, and a representative experiment is shown. The ability of the cells to adhere to a solid surface was significantly upregulated in the cells that had been treated with MTA1 siRNA. An asterisk (*) indicates a statistically significant difference (p<0.05) compared to the nontreated cells and negative-control siRNA-transfected cells. (C, D, and E) Using a Matrigel^TM^-coated transwell system, the invasive ability of the cells treated MTA1 siRNA was tested. The quantification of the number of invasive cells from the bottom of the transwell inserts is shown in the lower panel. An asterisk (*) indicates a statistically significant difference (p<0.05) compared with the nontreated cells and negative-control siRNA-transfected cells. (F, G, and H) The changes in the cytoskeleton structure were detected by confocal microscopy: Red staining represents α-tubulin. Blue staining represents the nuclei. The ‘feet’ were shortened, and the polarization was weakened in cells treated with MTA1 siRNA. These changes indicate a reduced ability to move (400 fold). A representative result from three independent experiments is shown for all data.

## Materials and Methods

### Antibodies and dyes

All reagents in this study were of analytical grade and are commercially available. The primary antibodies used in this study were purchased from the following companies: the MTA1 antibody was from Santa Cruz Biotech, USA; the E-cadherin antibody was from Epitomics Inc, USA; the α-tubulin and β-actin antibodies were from Sigma, Germany, and the p-AKT (ser473) antibody was from Cell Signaling Technology, USA. The alkaline phosphatase-conjugated anti-rabbit, anti-mouse or anti-goat IgGs were also purchased from Sigma. The FITC- and Cy3-conjugated IgGs were purchased from the PTG lab, USA. DAPI (4, 6-diamidino-2-phenylindole dihydrochloride, (2 μg/ml methanol)) was purchased from Taufkirchen, Germany. Wortmannin was also purchased from Sigma.

**Figure 3:**
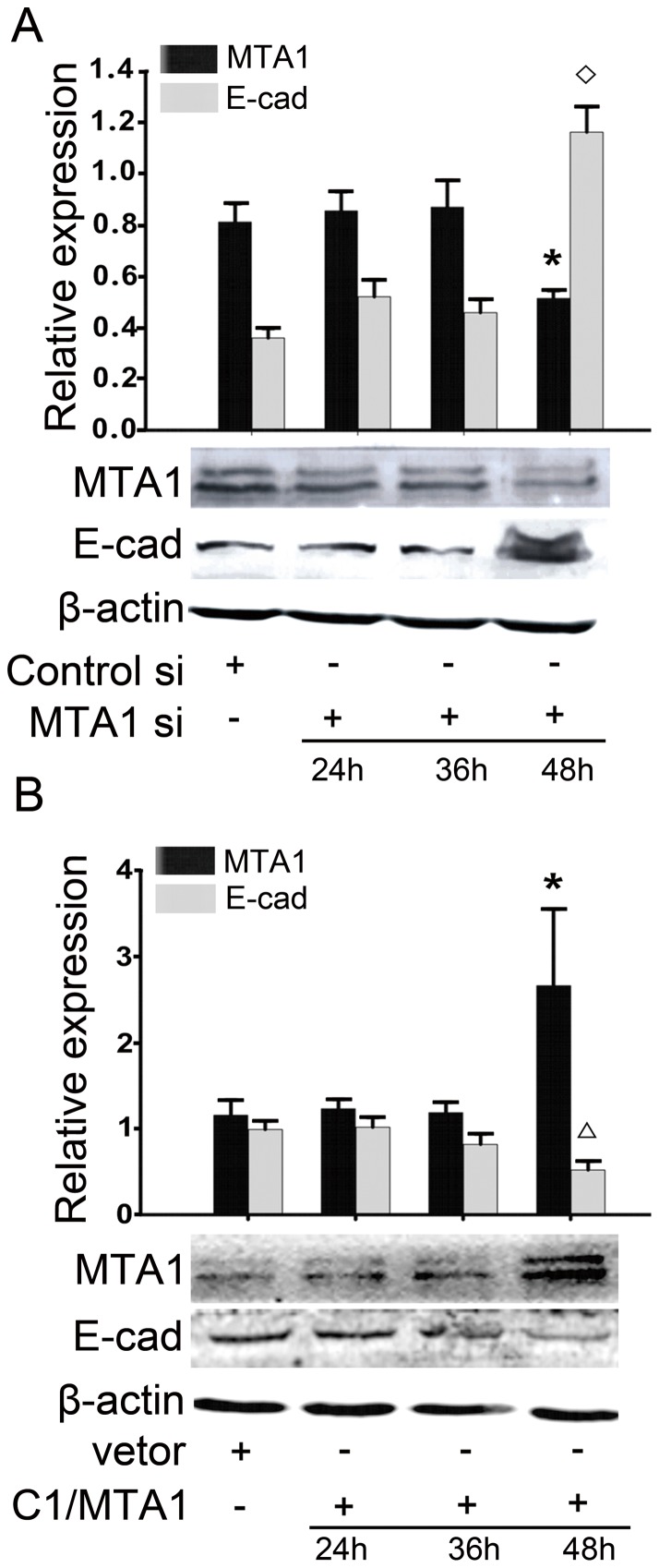
MTA1 regulates E-cadherin expression. (A) Western blotting results demonstrated that treatment with MTA1 siRNA increased E-cadherin expression after 48 hours. An asterisk (*) or diamond (◊) indicates a statistically significant difference (p<0.05) in the MTA1 or E-cadherin levels, respectively, compared with the negative-control siRNA-transfected cells. (B) A Western blotting analysis also demonstrated that transient transfection with a plasmid that encoded full-length MTA1 decreased E-cadherin expression after 48 hours. An asterisk (*) or diamond (◊) indicates a statistically significant difference (p<0.05) in the MTA1 or E-cadherin levels, respectively, compared with the cells transfected with an empty vector. The changes were quantified (top). All of the experiments were repeated three times.

### Tissue samples and cell lines

The tissues samples of normal prostate, localized prostate cancer and metastatic prostate cancer were obtained from the Department of Pathology of Tongji Hospital, which is affiliated with the Huazhong University of Science and Technology. All of the metastatic prostate cancer tissues were taken from patients who had documented metastatic disease and underwent a transurethral resection of the prostate (TURP) to relieve a urinary obstruction (distant metastasis and positive bone scan, M1 in tumor-node-metastasis TNM staging). This study was approved by local research ethics committee (REC) at the Tongji Hospital of Huazhong University of Science and Technology in accordance with the principle of the Helsinki Declaration II. All written informed consent documents from each participant were obtained during the enrollment phase. The poorly metastatic human prostate adenocarcinoma PC-3M-2B4 cell line (2B4) and the highly metastatic human prostate adenocarcinoma PC-3M-1E8 cell line (1E8) were purchased from Professor Zhengjie (Beijing University, China). The cells were cultured in RPMI 1640 medium (Gibco-BRL, US) with 10% (v/v) fetal bovine serum (Si Jiqing, Hangzhou, China).

**Figure 4:**
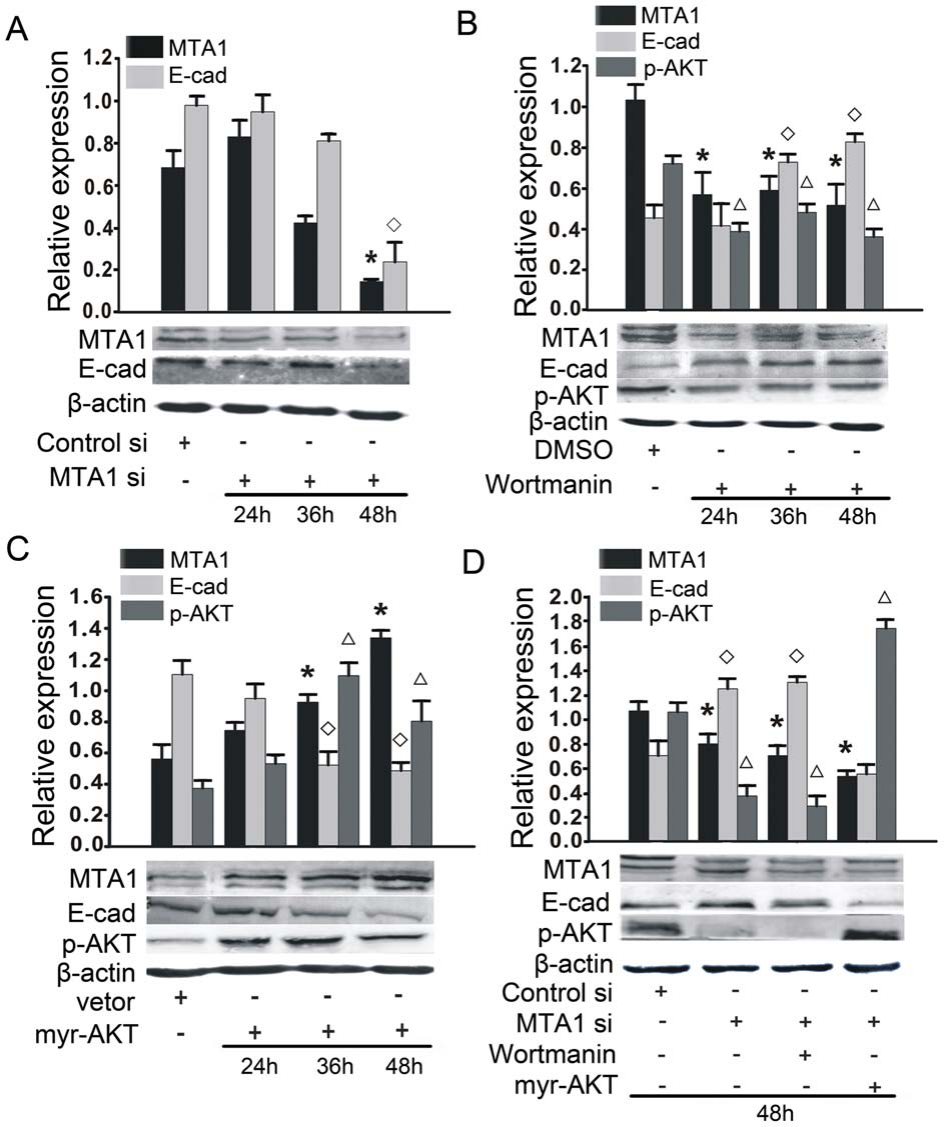
MTA1 regulates E-cadherin through the phosphorylation and activation of AKT (p-AKT). (A) A Western blotting analysis showed that treatment with MTA1 siRNA could inhibit AKT phosphorylation after a 48 hours siRNA treatment. An asterisk (*) and diamond (◊) indicates a statistically significant difference (p<0.05) in the MTA1 and E-cadherin levels, respectively, compared with the negative-control siRNA-transfected cells. (B) The cells were incubated with either the p-AKT inhibitor wortmannin (100 nM) or DMSO for various times from 24 hours to 48 hours, and the cellular proteins were extracted. A Western blotting analysis showed that wortmannin treatment promoted the expression of E-cadherin and inhibited the expression of MTA1 after 24 hours. An asterisk (*), diamond (◊), or triangle (Δ) indicates a statistically significant difference (p<0.05) in the MTA1, E-cadherin or p-AKT levels, respectively, compared with the DMSO-treated cells. (C) The forced over expression of p-AKT by transient transfection of the myr-AKT plasmid decreased the expression of E-cadherin and increased the expression of MTA1 after 24 hours. The quantification of the bands is shown (top). (D) The cells were transfected with MTA1 siRNA in the presence or absence of the myr-AKT plasmid or wortmannin for 48 hours. A Western blotting analysis was used to evaluate the changes in E-cadherin expression. An asterisk (*), diamond (◊), or triangle (Δ) indicates a statistically significant difference (p<0.05) in the MTA1, E-cadherin or p-AKT levels, respectively, compared with the negative-control siRNA-transfected cells. The quantification of the bands intensities is shown (top). All experiments were repeated three times.

### Immunohistochemistry

An immunohistochemical analysis of MTA1 was performed using the avidin-biotin-peroxidase complex method. Dewaxed and rehydrated tissue sections were incubated overnight at 4°C with a mouse monoclonal anti-human MTA1 antibody at a 1∶100 dilution and then washed with PBS. Biotinylated goat anti-rabbit immunoglobulin (DAKO, Kyoto, Japan) was then added to the sections for 30 minutes at room temperature. After the sections were washed with PBS, peroxidase-conjugated avidin (DAKO) was then applied. The peroxidase activity was detected by exposing the sections to a solution of 0.05% 3, 3-diaminobenzidine and 0.01% H_2_O_2_ in Tris-HCl buffer (3, 3-diaminobenzidine solution) for 3 to 6 minutes at room temperature. The sections were counterstained with hematoxylin.

**Figure 5:**
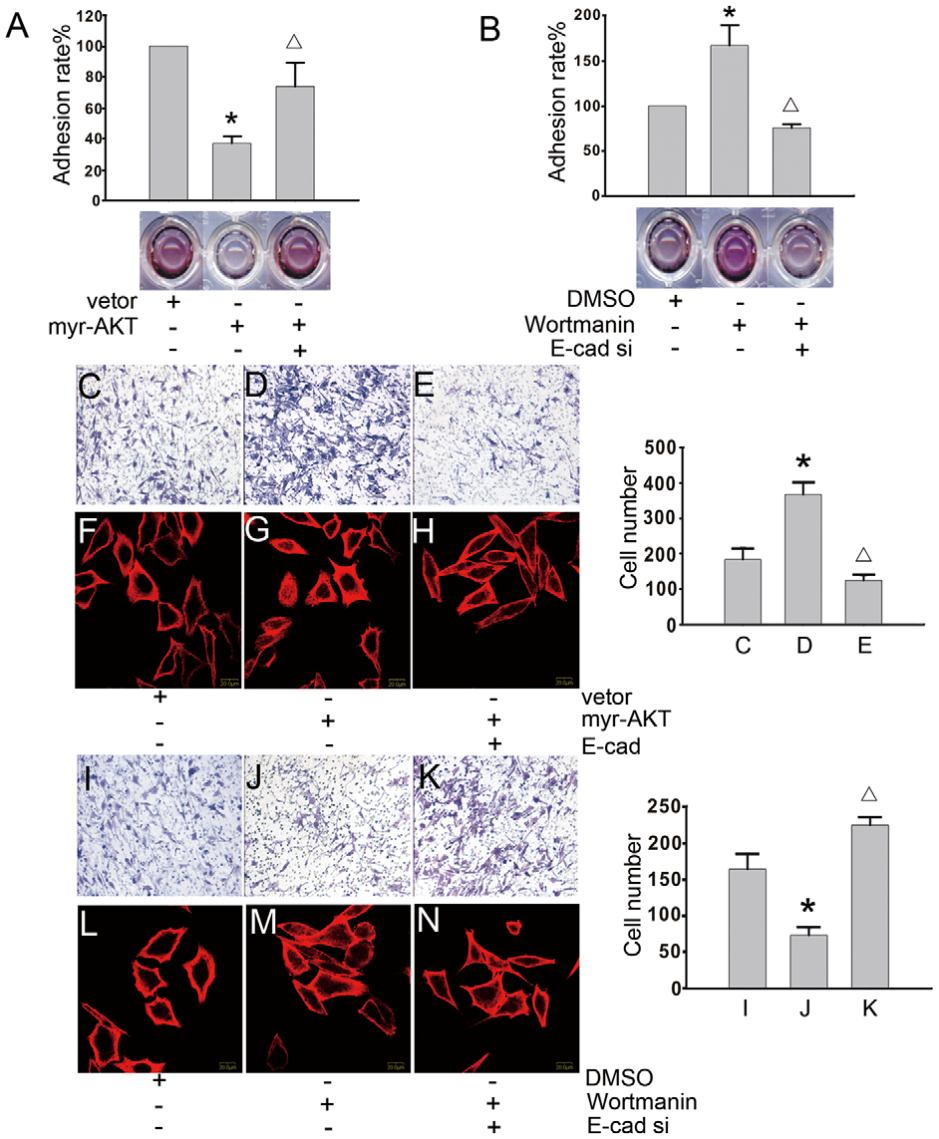
The forced over-expression or silencing of E-cadherin changes the p-AKT mediated malignant phenotypes. (A) The cells were treated with the myr-AKT plasmid or empty vector for 48 hours in the presence or absence of E-cadherin siRNA. The adhesive ability was tested using the MTT assay, and a typical experimental result is shown. An asterisk (*) indicates a statistically significant difference (p<0.05) compared to the cells transfected with an empty vector. A triangle (Δ) indicates a statistically significant difference (p<0.05) compared to the cells transfected with the myr-AKT-encoding plasmid in the absence of E-cadherin siRNA. (B) The cells were incubated with wortmannin (100 nM) or DMSO for 24 hours and then treated with or without E-cadherin siRNA for 48 hours. Using the MTT assay, the adhesive ability of the cells was tested. An asterisk (*) indicates a statistically significant difference (p<0.05) compared with DMSO-treated cells. A triangle (Δ) indicates a statistically significant difference (p<0.05) compared with the wortmannin-treated cells in the absence of E-cadherin siRNA. (C, D, and E) The cells were treated with the myr-AKT plasmid or an empty vector for 48 hours in the presence or absence of the E-cadherin plasmid. A Matrigel^TM^-coated transwell system was used to analyze the invasive capabilities of the cells. The quantification of the number of invasive cells from the bottom of the transwell inserts is shown (right panel). An asterisk (*) indicates a statistically significant difference (p<0.05) compared to the cells transfected with an empty vector. A triangle (Δ) indicates a statistically significant difference (p<0.05) compared with the cells transfected with a myr-AKT-encoding plasmid in the absence of E-cadherin siRNA. (F, G, and H) The cells were treated with the myr-AKT plasmid or an empty vector for 48 hours in the presence or absence of the E-cadherin plasmid. The changes in the cellular cytoskeleton were monitored by confocal microscopy. Red staining represents α-tubulin, and blue staining represents the nuclei (400 fold). (I, J, and K) The cells were treated with wortmannin (100 nM) or DMSO for 24 hours and then treated with or without E-cadherin siRNA for 48 hours. A Matrigel^TM^-coated transwell system was used to measure changes in the cellular invasive ability. The quantification of the number of invasive cells from the bottom of the transwell insert is shown (right panel). An asterisk (*) indicates a statistically significant difference (p<0.05) compared to the DMSO-treated cells. A triangle (Δ) indicates a statistically significant difference (p<0.05) compared with the wortmannin-treated cells in the absence of E-cadherin siRNA. (L, M, and N) The cells were treated with wortmannin (100 nM) or DMSO for 24 hours and then treated with or without E-cadherin siRNA for 48 hours. The changes in the cellular cytoskeleton were monitored by confocal microscopy. Red staining represents α-tubulin, and blue staining represents the nuclei (400 fold). A representative result from three experiments is shown for all data.

### RT-PCR and immunoblotting

For the RT-PCR analysis, total RNA was isolated from the cell lines using the Trizol reagent (Invitrogen, Cergy Pontoise, France), and the cDNA was synthesized with M-MLV reverse transcriptase (Promega, USA) according to the manufacturer's instructions. The PCR was performed using a PCR amplifying system (Biometra, USA). The primers were designed using Oligo 6 software, identified by the Basic Local Alignment Search Tool (BLAST; http://www.ncbi.nlm.nih.gov/BLAST) and purchased from Invitrogen. The MTA1 primer sequences were as follows: Forward: 5′- CTCTGCGCATCTTGTTGGACATA -3′ and Reverse: 5′-TCAGCTTCGTCGTGTGCAGATAG -3′. For an internal standard, the β-actin primer sequences were as follows: Forward: 5′- GCACCACACCTTCTACAATG -3′ and Reverse: 5′- TGCTTGCTGATCCACATC TG -3′.

For the Western blot analyses, the cells were lysed in RIPA buffer (50 mM Tris/HCl, pH 7.2, 150 mM NaCl, 1% NP-40, 0.1% SDS, and 0.5% (w/v) sodium deoxycholate). Equivalent amounts of the cell extracts (150 μg) were separated on a 10% sodium dodecyl sulfate polyacrylamide gel (SDS-PAGE) and transferred onto a polyvinylidene difluoride membrane (PVDF). The membranes were blocked in 25 mM Tris (pH 8.0) containing 125 mM NaCl, 0.1% Tween 20, and 5% skim milk for 1 hour and then incubated with the diluted primary antibodies (MTA1: 1∶200, E-cadherin and p-AKT: 1∶2000 and β-actin: 1∶1000) at 4°C overnight. After incubation with the primary antibodies, the secondary antibodies were added at a 1∶1000 dilution. The immunoreactive bands were visualized with alkaline phosphatase and BCIP/NBT staining.

### Quantitative Real-time PCR

For quantitative real-time PCR, total RNA was isolated from cell lines in Trizol reagent (Invitrogen, Cergy Pontoise, France). First-strand cDNA synthesis was performed using a cDNA synthesis kit (Amersham Bioscience, NJ). Quantitative real-time PCR was performed on an ABI PRISM 7700 Sequence Detection System (Applied Biosystems, CA) by using a QuantiTect SYBR Green kit (Qiagen, CA). The primers were designed using Primer Express 3.0 software, identified by Basic Local Alignment Search Tool (BLAST) (http://www.ncbi.nlm.nih.gov/BLAST) and purchased from Invitrogen. Each sample was run in triplicate. Conditions for quantitative PCR reaction were as follows, one cycle of 50°C for 15 min, one cycle of 94°C for 2 min, 40 cycles of 94°C for 15 s, 56°C for 20 s, and 70°C for 20 s. At the end of the PCR reaction, samples were subjected to a melting analysis to confirm specificity of the amplicon. The MTA1 primer sequences were: Forward: 5′- GCAGCTGAAGCTGAGAGCAAGTTA-3′; Reverse: 5′-CCTTGACGTTGTTGACGCTGA -3′. The E-cadherin primer sequences were: Forward: 5′- TACACTGCCCAGGAGCCAGA -3′; Reverse: 5′ TGGCACCAGTGTCCGGATTA-3′; For internal standard, the GAPDH primer sequences were: Forward: 5′- CCACTCCTCCACCTTTGAC-3′; Reverse: 5′- ACCCTGTTGCTGTAGCCA -3′.

### Cell transfection

The cell lines were cultured in 6-well tissue culture plates or flasks at 37°C with 5% CO2 in a humidified incubator (Heraeus, Germany). For the siRNA transfection, the cells were transfected with 200 pmol/ml of siRNA duplex using 5 μl of Lipofectamine^TM^ 2000 (Invitrogen) when the cells were 20% confluent according the manufacturer's protocol. The controls included untransfected cells and cells that had been transfected with a scrambled negative control siRNA (Ruibo, China). For the plasmid transfection, 4×10^5^ cells were plated in a 6-well plate and transfected using 4 μg of plasmid and 10 μl of Lipofectamine^TM^ 2000 per well. The full-length MTA1 plasmid (PEGFP-C1/MTA1) was kindly provided by Professor My Mahoney (Thomas Jefferson University), and the empty vector (PEGFP-C1) was preserved by our lab. The PCMV-SPORT6 E-cadherin plasmid was purchased from YRBIO (China), and the E-cadherin gene was subcloned into the pcDNA vector by our lab. The pcDNA myr-HA-AKT2 (myr-AKT) plasmid was purchased from Addgene (http://www.addgene.org/; Addgene plasmid 9016), and the empty vector pcDNA was also preserved in our lab. The cells were harvested 48 h after transfection, and the protein levels were measured by Western blot. The MTA1 siRNA sequences were as follows: Forward: 5′ CCCUGUCAGUCUGCUAUAA dTdT 3′ and Reverse: 3′ DTDTGGGACAGUCAGACGAUAUU 5′. The E-cadherin siRNA sequences were as follows: Forward: 5′CAGACAAAGACCAGGACUA dTdT 3′ and Reverse: 3′ dTdT GUCUGUUUCUGGUCCUGAU 5′.

### The invasion assay

For the invasion assay, 5×10^3^ cells were seeded in 100 μl RPMI 1640 media on the top of polyethylene terephthalate (PET) membranes coated with Matrigel^TM^ (1.5 mg/ml, BD Biosciences) within transwell cell culture inserts (24-well inserts, 8 μm pore size; Corning Life Sciences, Corning, NY). The bottom chamber was filled with 600 μl RPMI 1640 media containing 20% FBS and the supernatant from NIH3T3 cells (mouse embryonic fibroblast cell line) to act as a chemotactic factor (CF). The cells were incubated for 48 hours at 37°C with 5% CO_2_. Subsequently, the cells were fixed in 2.5% (v/v) glutaraldehyde and stained with crystal violet. The invasive cells on the gel bottom were visualized under a microscope (Leica, Germany) and quantified by counting the number of cells in three randomly chosen fields at a 100-fold magnification.

### The solid-phase adhesion assay

The adhesion assay was performed using the tetrazolium-based colorimetric assay (MTT). Equal numbers of cells (4×10^4^ cells per well) were seeded into 96-well plates that had been precoated with 1 μg/ml of fibronectin (FN) (Sigma, Germany). As a comparison, an equal number of cells was also seeded in plates coated with bovine serum albumin (1% w/v). After 1 hour, the plates were immersed into PBS containing 1 mM MgCl_2_ to remove non-adherent cells. Then, the number of adherent cells was measured with the MTT assay and read at 490 nm. The OD values reflected the proportion of cells that adhered to the FN-coated 96-well plate. The rate of adhesion was calculated by the following equation: the value of the OD of the experiment/the value of the OD of the control ×100%.

### Confocal microscopy imaging

For immunofluorescence staining, 1×10^4^ cells were seeded onto glass coverslips (13 mm diameter). After the cells were fixed with 4% paraformaldehyde in PBS, the cell membranes were permeabilized with 0.2% (v/v) Triton X-100 in PBS, and nonspecific binding sites were blocked with 5% BSA (w/v) in PBS. The first antibody was applied to the slides at 4°C overnight (α-tubulin: 1∶50, MTA1: 1∶20, and E-cadherin: 1∶100). After the incubation with the first antibody, the cells were washed three times with cold PBS and incubated with FITC- or Cy3-conjugated IgG at a 1∶50 dilution in PBS for 30 min at room temperature. The nuclei were stained for 5 min at room temperature with DAPI. The cells were rinsed with PBS and observed using a confocal microscope (Olympus, Japan).

### Co-immunoprecipitation(Co-IP)

1E8 cells grew in 10 cm dishes then were harvested in non-denaturing lysis buffer (20 mM Tris,, PH 8, 135 mM NaCl, 10% glycerol, 1%Nonidet P-40 (NP-40), 2 mM EDTA, Roche complete mini protease cocktail inhibitors. All of the following procedures were performed on the ice. Lysis buffer-soluble lysates, collected following extraction for 4 hours and centrifugation at 12,000 rpm 20 minutes at 4°C. In a tube 1mg cell lysate was added with 40 μl MTA1 antibody, and incubated overnight at 4°C. The next day, cell lysate containing antibody were applied to protein G-coupled sepharose beads (Abcam) for 4 hours at 4°C. Beads were washed 3 times in lysis buffer. Finally, the supernatant were added into 25μl 2× loading buffer. Western blotting was performed after separating protein and beads with boiling.

### Statistical analysis

The data were analyzed by an ANOVA. The statistical analyse was performed using SPSS version 13.0 software.

## Results

### The expression of MTA1 in prostate carcinoma

To confirm the expression of MTA1 in human prostate cancer, an immunohistochemical staining analysis for MTA1 was performed on normal prostate, localized prostate cancer and metastatic prostate cancer tissue samples (the resources and criteria for tissue designations are described in the materials and methods section). The results revealed that in normal prostate samples, MTA1 expression was barely detectable (Figure 1A). However, in the localized prostate cancer samples, positive staining for MTA1 was observed in the nuclei of 6 of the 15 tissues examined (Figure 1B). Additionally, in 27 of the 30 tumor metastatic prostate cancer tissues, a high level of MTA1 staining was observed in both the cytoplasm and nuclei of the cancerous cells (Figure 1C). A quantitative analysis for the positive staining of MTA1 in Figure 1A–C was shown in Figure S1. An RT-PCR and Western blot analysis were performed to measure the expression of MTA1 RNA and protein levels, respectively, in PC-3M-1E8 (1E8) and PC-3M-2B4 (2B4) cells [29], [30]. The results from these analyses are shown in Figure 1D and E, which demonstrate that MTA1 RNA and protein levels were present in both cell lines but at different expression levels. The mRNA expression levels of MTA1 were 0.38±0.01 and 0.83±0.02 for the 2B4 and 1E8 cells, respectively, and the protein expression levels in the 2B4 and 1E8 cells were 0.58±0.04 and 0.83±0.02, respectively (p<0.05, n = 3 for each). We found that the 1E8 cells expressed approximately two-fold higher levels of MTA1 RNA and 1.5-fold higher MTA1 protein levels compared to the 2B4 cells. Therefore, the 1E8 cell line was chosen to investigate the biological properties of MTA1 in prostate carcinoma in vitro.

### The influence of MTA1 silencing on the malignant phenotype of 1E8 cells

We used siRNA to down-regulate the expression of MTA1 in the 1E8 cells. Three pairs of siRNAs against MTA1 were designed. We verified the efficacy of the siRNA by Western blotting (Figure S2). At least a 60% knockdown of the MTA 1 protein level was obtained using the siRNA-3#, so we chose this pair for our further study (Figure 2A). We also over-expressed MTA1 in the background of MTA1 siRNA, which confirmed the effect brought by siRNA-3# in cells (Figure S3). The siRNA-transfected cells were also analyzed for the expression of the E-cadherin protein. A Western blot analysis showed the expression level of E-cadherin was higher in the MTA1 siRNA-treated cells (48 hours after transfection), which indicated that MTA1 may play a role in the negative regulation of E-cadherin. Next, we analyzed the adhesion ability of the MTA1 siRNA-treated 1E8 cells using a solid-phase assay combined with the MTT assay (see the materials and methods section). For the same surface-coated concentration of FN, the adhesion ability of the MTA1 siRNA-treated cells was significantly higher than the control cells. A representative image is shown. The adhesive rates for the nontreated, negative control siRNA-treated and MTA1 siRNA-treated cells were 40.07±6.23, 50.22±4.99 and 99.62±9.62, respectively (p<0.05, n = 3, Figure 2B). We also studied the effects of MTA1 on the invasiveness of the 1E8 cells using a Matrigel^TM^-coated transwell model. The quantities of cells at the bottom of the membrane, which reflected the invasiveness of the cells were 597±6.24, 590±5.77 and 201±2.40 for the nontreated, negative control siRNA-treated and MTA1 siRNA-treated cells, respectively (p<0.05, n = 3 Figure 2C–E). Alterations of the cellular cytoskeleton organization and polarity are also involved in tumor cell metastasis [31] Therefore, we examined the cytoskeleton structures of the MTA1 siRNA-treated cells by confocal microscopy and found that the ‘feet’ of the MTA1 siRNA-treated cells were shortened and that the cellular polarity was weakened compared to the control cells (Figure 2F-H MTA1: red; nuclei: blue). These results further suggest that MTA1 may be involved in the malignant phenotypes of cancer cells, including adhesion, invasiveness, cytoskeletal structure and cell polarity.

### MTA1 regulates the expression of E-cadherin

To confirm the role of MTA1 in the regulation of E-cadherin expression, we treated 1E8 cells with MTA1 siRNA and a full-length MTA1 plasmid for various times from 24 to 48 hours (translational level). As shown in Figure 3A, after 48 hours of MTA1 siRNA treatment, the expression of MTA1 was down-regulated, and the expression of E-cadherin was significantly upregulated The protein expression level of E-cadherin was 1.16±0.10 compared to 0.35±0.04 in the control cells (p<0.05, n = 3). Then, we tested the expression level of E-cadherin in 1E8 cells that had been transfected with the pEGFP-C1/MTA1 plasmid that encoded the full-length MTA1 gene (Figure 3B). Compared with cells that had been transfected with the empty pEGFP-C1 vector, the expression of E-cadherin was significantly down-regulated in the cells over-expressing MTA1. The protein expression level of E-cadherin was 0.25±0.08 in the cells over-expressing MTA1 compared to 1.08±0.36 in the control cells (p<0.05, n = 3). The same tendency was also detected by realtime-PCR in RNA level (Figure S4).

### MTA1 regulates E-cadherin expression through AKT activation

Based on the previously mentioned literature, we investigated the potential role of p-AKT in the interaction between MTA1 and E-cadherin. We found that MTA1 siRNA treatment significantly reduced the level of p-AKT after 48 hours (Figure 4A). We also treated cells with wortmannin (100 nM), a PI3K inhibitor, which also led to a decrease in the level of AKT phosphorylation. The selective inhibition of p-AKT resulted in a significant upregulation of E-cadherin expression and downregulation of MTA1 expression after 36 hours or 48 hours (Figure 4B). Similarly, transient transfection with the pcDNA3 myr-HA-AKT2 plasmid (myr-AKT) resulted in an activation of p-AKT, decreased the expression of E-cadherin and increased the expression of MTA1 after 36 hours or 48 hours (Figure 4C). Considering the different timing of the interactions between MTA1, p-AKT and E-cadherin, we chose 48 hours as the timepoint for subsequent experiments. We found that the increased E-cadherin protein expression caused by MTA1 siRNA treatment was further enhanced by wortmannin treatment (100 nM) and blocked upon transfection with the myr-AKT plasmid (Figure 4D). Cumulatively, these results demonstrate that the down-regulation of MTA1 induced E-cadherin expression through the activation of AKT, which indicates that p-AKT serves a mediator between the two genes and plays an important role in the malignant phenotypes regulated by MTA1 in 1E8 cells. All of the experiments were repeated three times.

### Changes in the expression of E-cadherin alter the p-AKT-mediated cellular malignant phenotype

Based on the results shown in Figures 3 and 4, we hypothesized that MTA1 may regulate E-cadherin through p-AKT to induce the invasion of 1E8 cells. Therefore, increasing or decreasing the expression of E-cadherin may alter the cellular malignant phenotype mentioned in Figure 2. To explore this possibility, we examined whether changes in E-cadherin expression altered the p-AKT-induced cellular adhesion. As shown in Figure 5A, cells that had been transfected with myr-AKT had a lower adhesive ability compared to cells transfected with an empty vector. Additionally, the co-transfection of myr-AKT and E-cadherin increased the adhesive ability of the cells. In contrast, treatment with E-cadherin siRNA inhibited the enhanced adhesion ability caused by wortmannin treatment (100 nM) (Figure 5B). Next, we examined if expression levels changes in E-cadherin were able to alter the invasiveness of the cells or change the cellular cytoskeleton, which are mediated by p-AKT. As shown in Figure 5 C, D and E, using a Matrigel^TM^-coated transwell system (see the actual number of cells on the right), we found that the forced over-expression of E-cadherin inhibited the ability of myr-AKT to enhance cell invasion. Additionally, using confocal microscopy, we found that the ‘feet’ of cells over-expressing myr-AKT were longer and that the polarity of the cells was increased, while E-cadherin over-expression shortened the ‘feet’ and reversed the polarity (Figure 5F, G, and H). In contrast, the invasion of wortmannin-treated (100 nM) cells was inhibited, and the down-regulation of E-cadherin expression by siRNA treatment enhanced the invasion (Figure 5I, J, and K) (see the number cells within each panel). As shown in Figure 5L, M, and N, the cells that had been treated with a combination of E-cadherin siRNA and wortmannin (100 nM) had longer ‘feet’ and sharper polarity compared to cells that had been treated with wortmannin (100 nM) or DMSO alone. All of the experiments were repeated three times.

## Discussion

The MTA1 gene has been found to be associated with many tumors, including esophageal carcinoma [32], thymoma [33], ovary cancer [34], and breast cancer [35]. All of these studies have indicated that MTA1 plays an important role in tumor cell invasion and metastasis. Thus, the MTA1 gene has been considered to be good target for overcoming tumor metastasis and invasion. In prostate cancer, a DNA microarray demonstrated that MTA1 is selectively over-expressed in metastatic prostate cancer compared to clinically localized prostate cancer or benign prostate tissue [4], [36]. Little is known regarding how MTA1 functions to regulate metastasis in prostate cancer. The work presented in this study suggests that the regulation of E-cadherin by MTA1 may be an important step for cellular transformation, and p-AKT serves as the mediator for this regulation in prostate cancer cells. Our results support this conclusion with a detailed analysis from the tissue level to the molecular level. An immunohistochemistry analysis demonstrated that MTA1 was over-expressed in prostate cancer tissues, especially in metastatic prostate cancer tissue. The protein and mRNA levels of MTA1 were significantly higher in the highly metastatic prostate cancer PC-3M-1E8 cells than in the poorly metastatic prostate carcinoma PC-3M-2B4 cells. Our results also indicate that the MTA1 gene may play an important role in the invasion and metastasis of prostate cancer. To elucidate the mechanism of MTA1 in prostate cancer metastasis, we chose the metastatic prostate cancer PC-3M-1E8 cell line that contained a high level of MTA1 for subsequent analysis.

We found that after the MTA1 gene was down-regulated by siRNA treatment, the malignant phenotypes of the 1E8 cells were inhibited, including increased cellular adhesion, decreased cellular invasiveness and weakened polarity of the cellular cytoskeleton. The alteration of the malignant phenotypes of cells is one of the most typical features of the EMT, and E-cadherin has been shown to play a crucial role in the EMT [37]. MTA1 has also been shown to regulate the EMT pathway at multiple points, including E-cadherin expression [38]. E-cadherin is one of the most important molecules that controls the properties of the cellular malignant phenotype, such as adhesion, invasion, and filament systems [39], [40], [41]. As shown in Figure 2, the silencing of MTA1 increased the expression of E-cadherin in 1E8 cells, which was consistent with our previous study [22]. Based on the aforementioned data and literature, we hypothesized that the regulation of the malignant phenotype of 1E8 cells was related to the regulation of E-cadherin expression by MTA1.

Then, we examined the regulation of E-cadherin by MTA1 at various times after transfection. As shown in Figure 3, the down-regulation of MTA1 expression by siRNA treatment selectively enhanced the expression of E-cadherin 48 hours after transfection, while the over-expression of MTA1 inhibited E-cadherin expression 48 hours after transfection. From these data, we chose the 48 hours timepoint for subsequent experiments.

The subcellular location of proteins is closely related to protein function and provides a potential type of interaction [42]. Previous reports have shown that MTA1 was expressed exclusively in the nucleus [43] and cytoplasm [44] and that E-cadherin was expressed mainly on the cell surface [45]. We did not examine a transmembrane region in the MTA1 gene [5], and we can not pull E-cadherin down by MTA1 antibody (Figure S5), so we hypothesized that a mediator may facilitate the interaction between MTA1 and E-cadherin.

Interestingly, among the MTA1-interacting proteins, we identified phosphorylated AKT, which could potentially regulate E-cadherin expression as cross talk between PI3K/AKT, MTA1 and E-cadherin has been implied [28], [46], [47]. Additionally, AKT signaling has been shown to be a key controller of tumor cell metastasis and proliferation [48], [49]. These studies led us to investigate whether p-AKT was essential for the MTA1-mediated regulation of E-cadherin expression in 1E8 cells. We found that the down-regulation of MTA1 expression by siRNA treatment led to a down-regulation of p-AKT expression. Alterations in p-AKT expression by incubation with a p-AKT inhibitor or by over-expression of the myr-AKT plasmid resulted in changes in the expression levels of MTA1 and E-cadherin. Increasing the expression of p-AKT inhibited the upregulation of E-cadherin expression by MTA1, which was enhanced by decreasing the expression of p-AKT. These data suggest that MTA1 regulates E-cadherin expression in a p-AKT-dependent manner.

Clearly, the data presented in this study demonstrate that E-cadherin expression is controlled by MTA1 through a p-AKT-dependent mechanism; therefore, alterations in the expression of E-cadherin may block the ability of p-AKT to control the malignant phenotype of cancerous cells. Altered expression of p-AKT by incubation with a p-AKT inhibitor or transfection with a plasmid encoding myr-AKT changed the malignant phenotypes of cells, including the adhesive ability, invasiveness and polarity of the cells. Changes in the expression of E-cadherin caused by transfection with an E-cadherin-encoding plasmid or siRNA treatment also modified the p-AKT-mediated malignant transformation. These results are consistent with a recent report that demonstrated that E-cadherin directly contributed to PI3K/AKT activation in ovarian carcinoma cells [50].

Taken together, these findings suggest the existence of a new MTA1-dependent pathway, the MTA1/p-AKT/E-cadherin pathway, which controls the malignant phenotype in prostate cancer cells. A better understanding of the mechanisms of metastasis genes like MTA1 provides greater insight into the metastasis of prostate carcinoma as well as a potential therapeutic target for the treatment of metastasis.

## Supporting Information

Figure S1 **Quantitative analysis for the positive staining of MTA1 in** **Fig. 1** **(A–C).**
(TIF)

Figure S2 **Silencing MTA1 expression by siRNAs transfection in 1E8 cells.** Three pairs of siRNA (1#, 2#, 3#) and negative control siRNA were transfected for 48h. The protein expression of MTA1 and E-cadherin was analysis by western blotting, and β-actin was used for a loading control. An asterisk (*) or diamond (◊) indicates a statistically significant difference (p<0.05) in the MTA1 or E-cadherin levels, respectively, compared with the negative-control siRNA-transfected cells, n = 3.
(TIF)

Figure S3 **Suppressing RNAi effect by MTA1 overexpression.** Western blotting results demonstrated that transfecting MTA1 full length plasmid suppressed RNAi effect brought by MTA1 siRNA treatment. An asterisk (*)indicates a statistically significant difference (p<0.05) in the MTA1 levels, compared with the negative-control siRNA-transfected cells, n = 3.
(TIF)

Figure S4 **MTA1 regulates E-cadherin expression in mRNA level.** (A) Quantitative PCR (qPCR) analysis of MTA1 and E-cadherin mRNA expression in 1E8 cells after treatment with MTA1 siRNA after 24, 36 and 48 hours. An asterisk (*) or diamond (◊) indicates a statistically significant difference (p<0.05) in the MTA1 or E-cadherin levels, respectively, compared with the negative-control siRNA-transfected cells.(B) qPCR also analysis of E-cadherin mRNA and MTA1 expression in 1E8 cells after transfection with a plasmid that encoded full-length MTA1 decreased E-cadherin expression after 24, 36 and 48 hours. An asterisk (*) or diamond (◊) indicates a statistically significant difference (p<0.05) in the MTA1 or E-cadherin levels, respectively, compared with the cells transfected with an empty vector. The changes were quantified. All of the experiments were repeated three times.
(TIF)

Figure S5 **MTA1 and E-cadherin had indirect regulation.** 1E8 protein was incubated with MTA1 antibody, and E-cadherin protein did not be pulled down by co-immunoprecipitation assay. The assay was repeated for 4 times.
(TIF)
