# Expression of Concern: Akt Mediates Metastasis-Associated Gene 1 (MTA1) Regulating the Expression of E-cadherin and Promoting the Invasiveness of Prostate Cancer Cells

**DOI:** 10.1371/journal.pone.0266930

**Published:** 2022-04-07

**Authors:** The PLOS ONE Editors

After this article [1] was published, concerns were raised about similarities between figures within the article and also between figures in this article [1] and a previously published work [2].

Specifically:

- Fig 2E in this article [1] appears similar to the Mock panel in Fig 3C in [2] (subsequently corrected in [3]).
- Fig 5J in this article [1] appears similar to the Control siRNA panel in Fig 3C in [2] (subsequently corrected in [3]).
- In Fig 3A in this article [1], the MTA1 panel appears similar to the MTA1 panel in Fig 4A. The MTA-1 panel in Fig 4A is incorrect.
- In Fig 5A in this article [1], the MTT assay well images on the left and right hand sides appear similar to each other. Both well images are incorrect.
- In S2 Fig in this article [1], the β-actin blot panel appears similar to the β-actin blot panel in S3 Fig.
- Lanes 1–3 in the β-actin blot panels in S2 and S3 Figs appear similar to the β-actin blot panel in Fig 4B in [2] when re-sized vertically and horizontally.

One of the corresponding authors of the article [1] contacted *PLOS ONE* to request correction of errors in Figs 4 and 5. In response to the journal’s follow up on the above issues, a corresponding author stated that errors in Figs 4A and 5A occurred during the preparation of the manuscript due to the mislabeling of images and folders, and that the E-cad panel in Fig 4A is mislabeled. They have provided an updated version of Fig 4 (S1 File), in which the three blots and associated chart in Fig 4A have been replaced, and the E-cad label corrected to p-AKT, and an updated version of Fig 5 (S2 File), in which the left and right MTT assay well images in Fig 5A have been replaced. Underlying data to support the chart and blots in the revised Fig 4A and the left and right MTT assay well images in the revised Fig 5A are provided in S3–S5 Files. The corresponding author indicated that these errors do not affect the results and conclusions of the experiments.

The Mock and Control siRNA panels in Fig 3C in [2] have been replaced in an Erratum [3] which removes the similarities with Figs 2E and 5J of this article [1].

The authors did not respond to the queries about S2 and S3 Figs.

Underlying data to support Fig 2E, Fig 3A and the remainder of Figs 4 and 5 were requested in editorial follow up but have not been provided. The *PLOS ONE* Editors were unable to obtain further methodological information regarding the western blot experiments, including whether total AKT was measured as a control for phosphor-AKT.

The related study published in [2] was not cited and discussed in [1]. An Academic Editor reviewed articles [1] and [2] and noted that whilst they address broadly the same research question, there are differences in methodology and results, and this article [1] identifies a specific intracellular pathway (AKT) responsible for phenotypic changes. They also noted that details on how the quantification in Fig 4 was carried out have not been provided nor confirmation that the cell viability (MTT) assay data is represented quantitatively as a mean from multiple readings.

Due to the concerns raised about image and data handling practices that may affect the reliability of published results, the unresolved concerns regarding S2–S3 Figs, and the unavailability of some underlying data, the *PLOS ONE* Editors issue this Expression of Concern.

## Supporting information

S1 File A revised version of Fig 4. (TIF)

S2 File A revised version of Fig 5. (TIF)

S3 File Quantitative data underlying the revised Fig 4A. (XLS)

S4 File Primary data underlying the revised Fig 4A blots. (TIF)

S5 File Primary data underlying the revised Fig 5A MTT assay. (JPG)
