# Supplementary figures and images for: Akt Mediates Metastasis-Associated Gene 1 (MTA1) Regulating the Expression of E-cadherin and Promoting the Invasiveness of Prostate Cancer Cells
Source: PLoS One. 2012 Dec 5;7(12):e46888. doi: 10.1371/journal.pone.0046888 (PMC3515600; doi:10.1371/journal.pone.0046888)

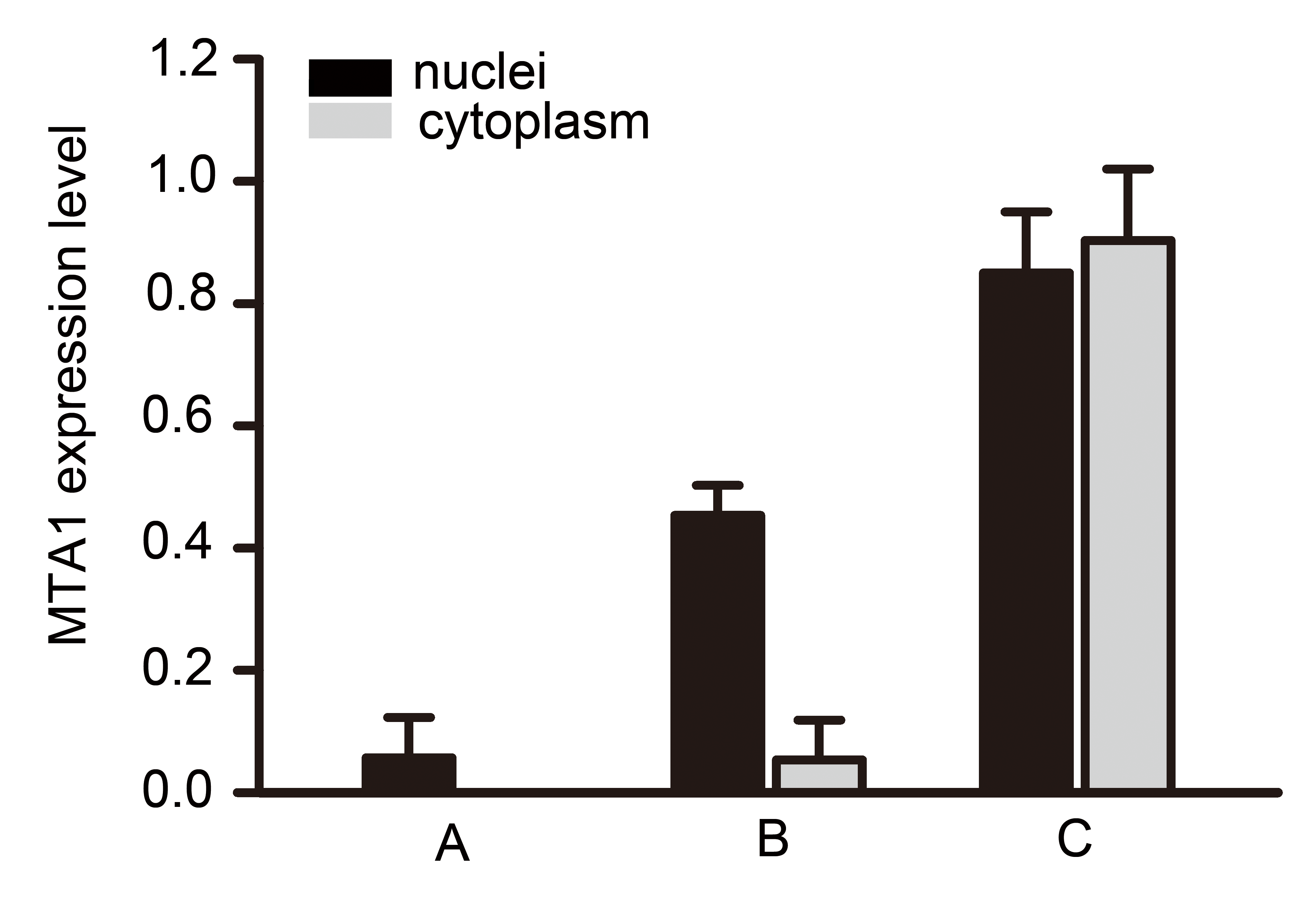

Supplement: Figure S1 — Quantitative analysis for the positive staining of MTA1 in Fig. 1 (A–C). (TIF) [file pone.0046888.s001.tif]

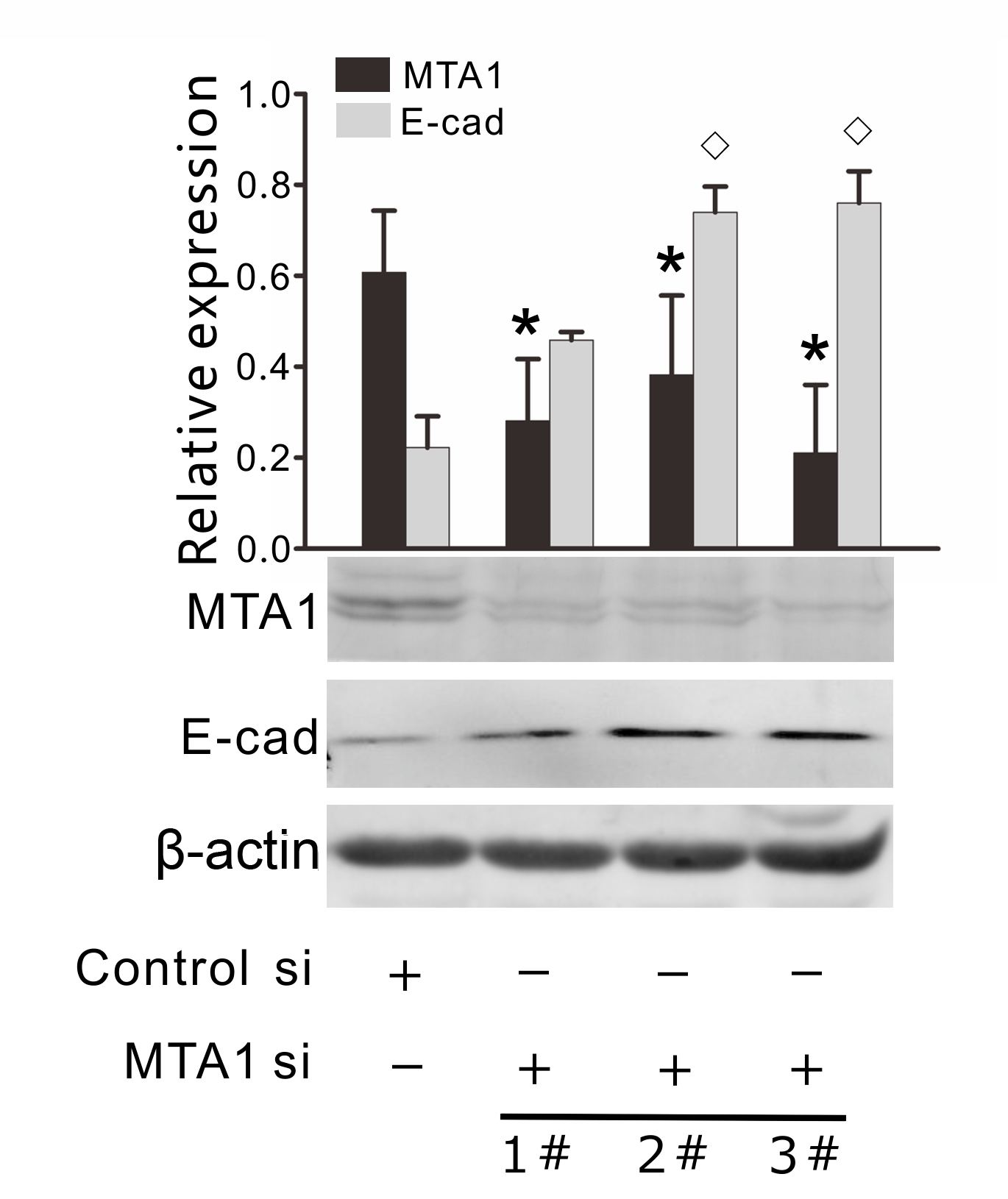

Supplement: Figure S2 — Silencing MTA1 expression by siRNAs transfection in 1E8 cells. Three pairs of siRNA (1#, 2#, 3#) and negative control siRNA were transfected for 48h. The protein expression of MTA1 and E-cadherin was analysis by western blotting, and β-actin was used for a loading control. An asterisk (*) or diamond (◊) indicates a statistically significant difference (p<0.05) in the MTA1 or E-cadherin levels, respectively, compared with the negative-control siRNA-transfected cells, n = 3. (TIF) [file pone.0046888.s002.tif]

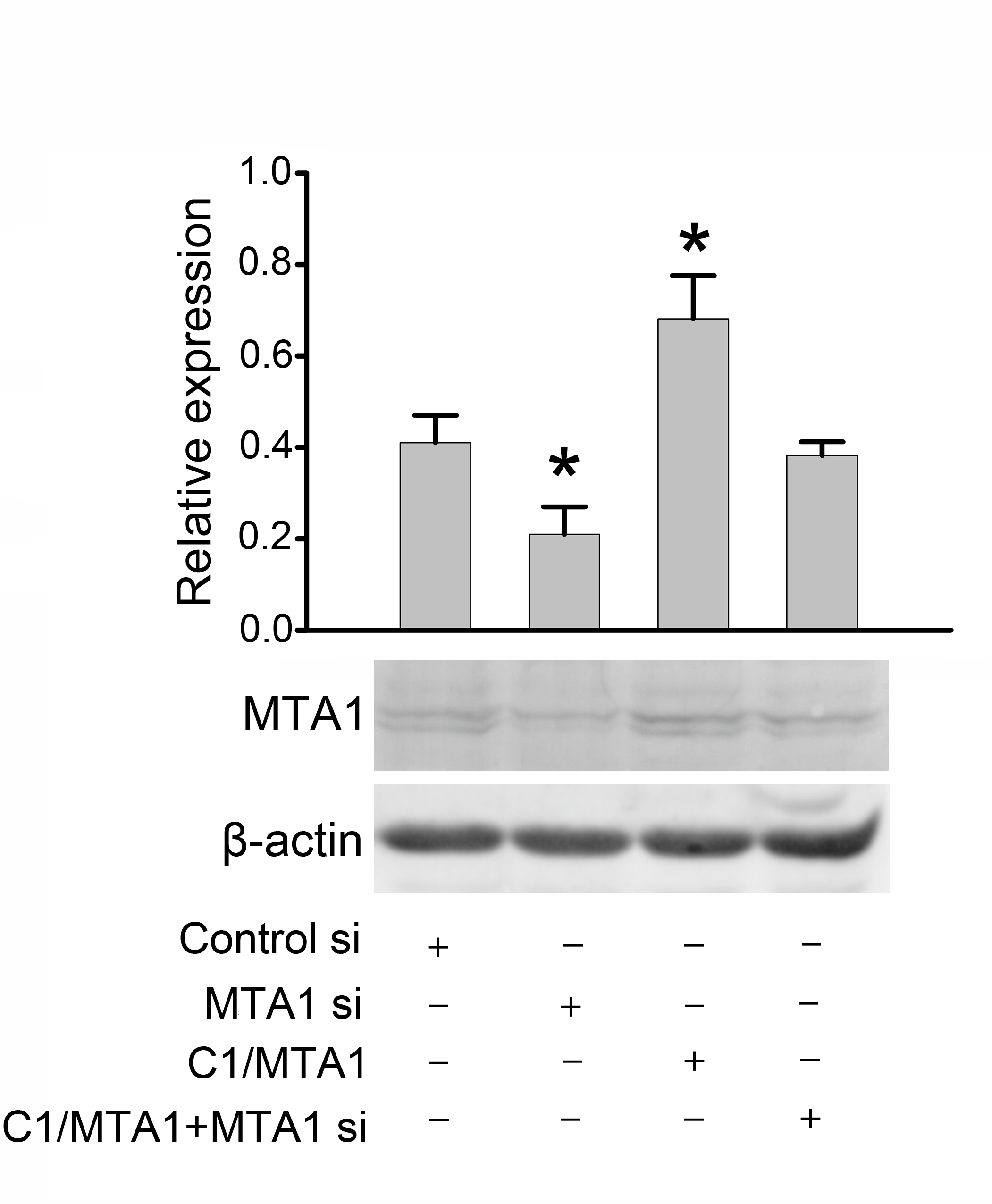

Supplement: Figure S3 — Suppressing RNAi effect by MTA1 overexpression. Western blotting results demonstrated that transfecting MTA1 full length plasmid suppressed RNAi effect brought by MTA1 siRNA treatment. An asterisk (*)indicates a statistically significant difference (p<0.05) in the MTA1 levels, compared with the negative-control siRNA-transfected cells, n = 3. (TIF) [file pone.0046888.s003.tif]

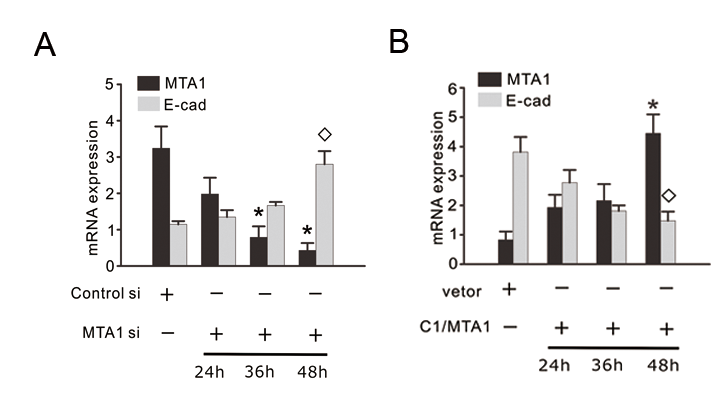

Supplement: Figure S4 — MTA1 regulates E-cadherin expression in mRNA level. (A) Quantitative PCR (qPCR) analysis of MTA1 and E-cadherin mRNA expression in 1E8 cells after treatment with MTA1 siRNA after 24, 36 and 48 hours. An asterisk (*) or diamond (◊) indicates a statistically significant difference (p<0.05) in the MTA1 or E-cadherin levels, respectively, compared with the negative-control siRNA-transfected cells.(B) qPCR also analysis of E-cadherin mRNA and MTA1 expression in 1E8 cells after transfection with a plasmid that encoded full-length MTA1 decreased E-cadherin expression after 24, 36 and 48 hours. An asterisk (*) or diamond (◊) indicates a statistically significant difference (p<0.05) in the MTA1 or E-cadherin levels, respectively, compared with the cells transfected with an empty vector. The changes were quantified. All of the experiments were repeated three times. (TIF) [file pone.0046888.s004.tif]

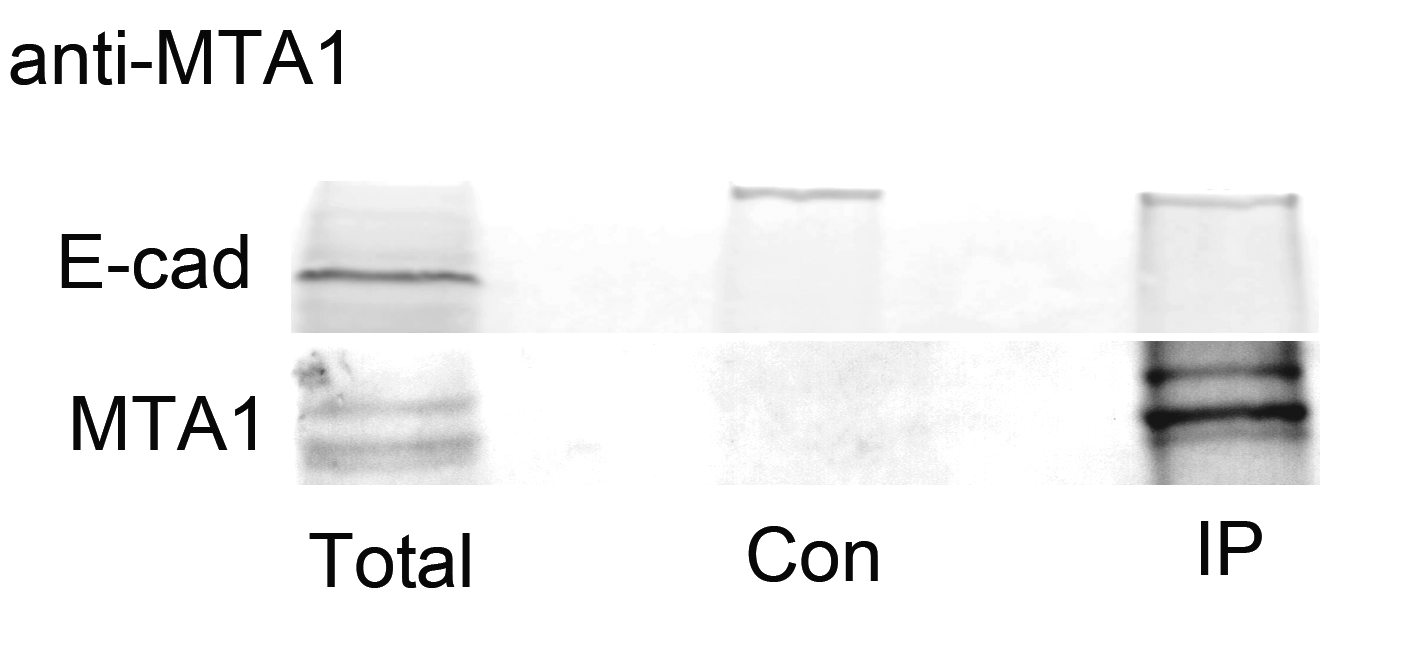

Supplement: Figure S5 — MTA1 and E-cadherin had indirect regulation. 1E8 protein was incubated with MTA1 antibody, and E-cadherin protein did not be pulled down by co-immunoprecipitation assay. The assay was repeated for 4 times. (TIF) [file pone.0046888.s005.tif]
