# Supplementary figures and images for: Expression of Concern: Akt Mediates Metastasis-Associated Gene 1 (MTA1) Regulating the Expression of E-cadherin and Promoting the Invasiveness of Prostate Cancer Cells
Source: PLoS One. 2022 Apr 7;17(4):e0266930. doi: 10.1371/journal.pone.0266930 (PMC8989188; doi:10.1371/journal.pone.0266930)

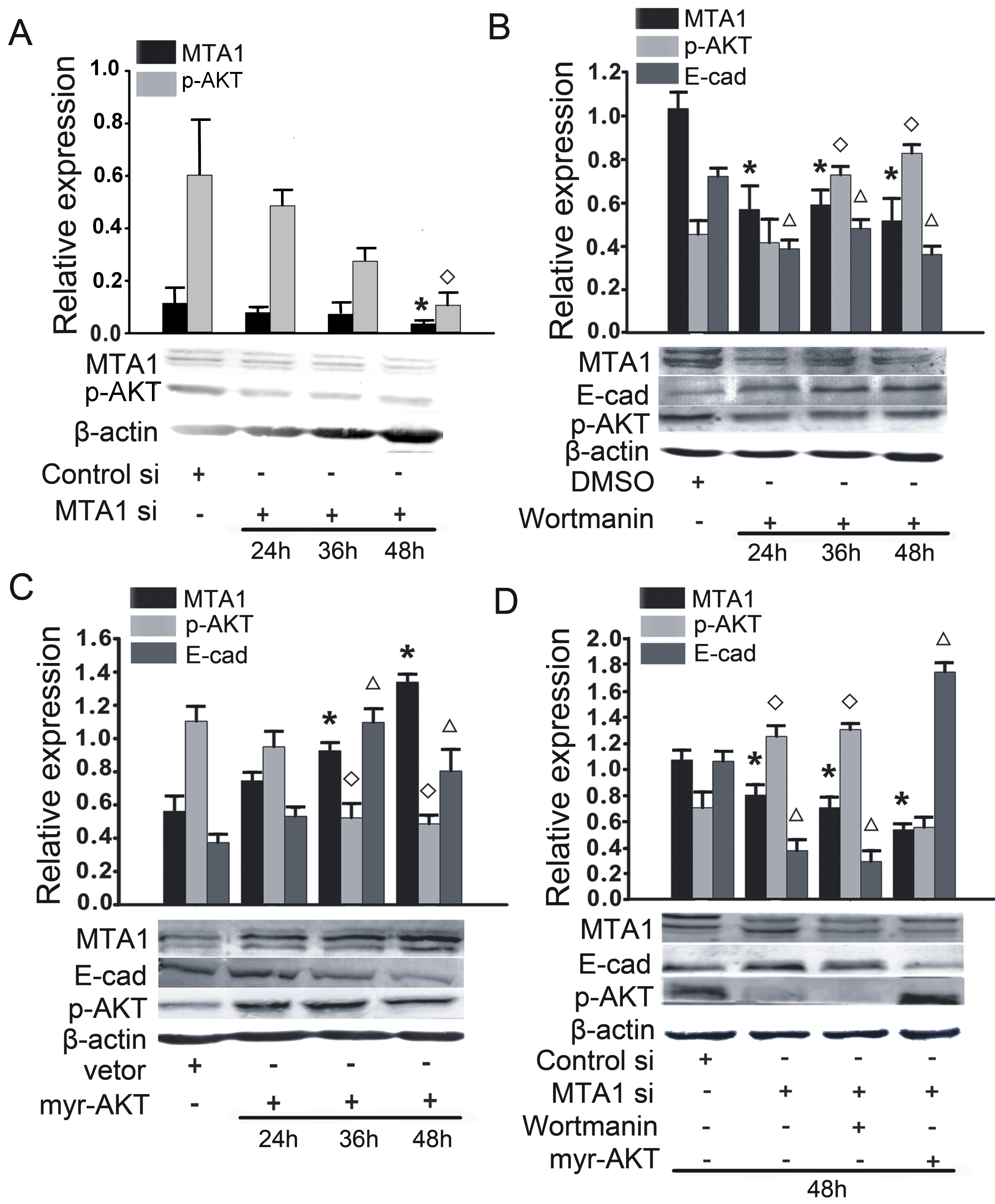

Supplement: S1 File — (TIF) [file pone.0266930.s001.tif]

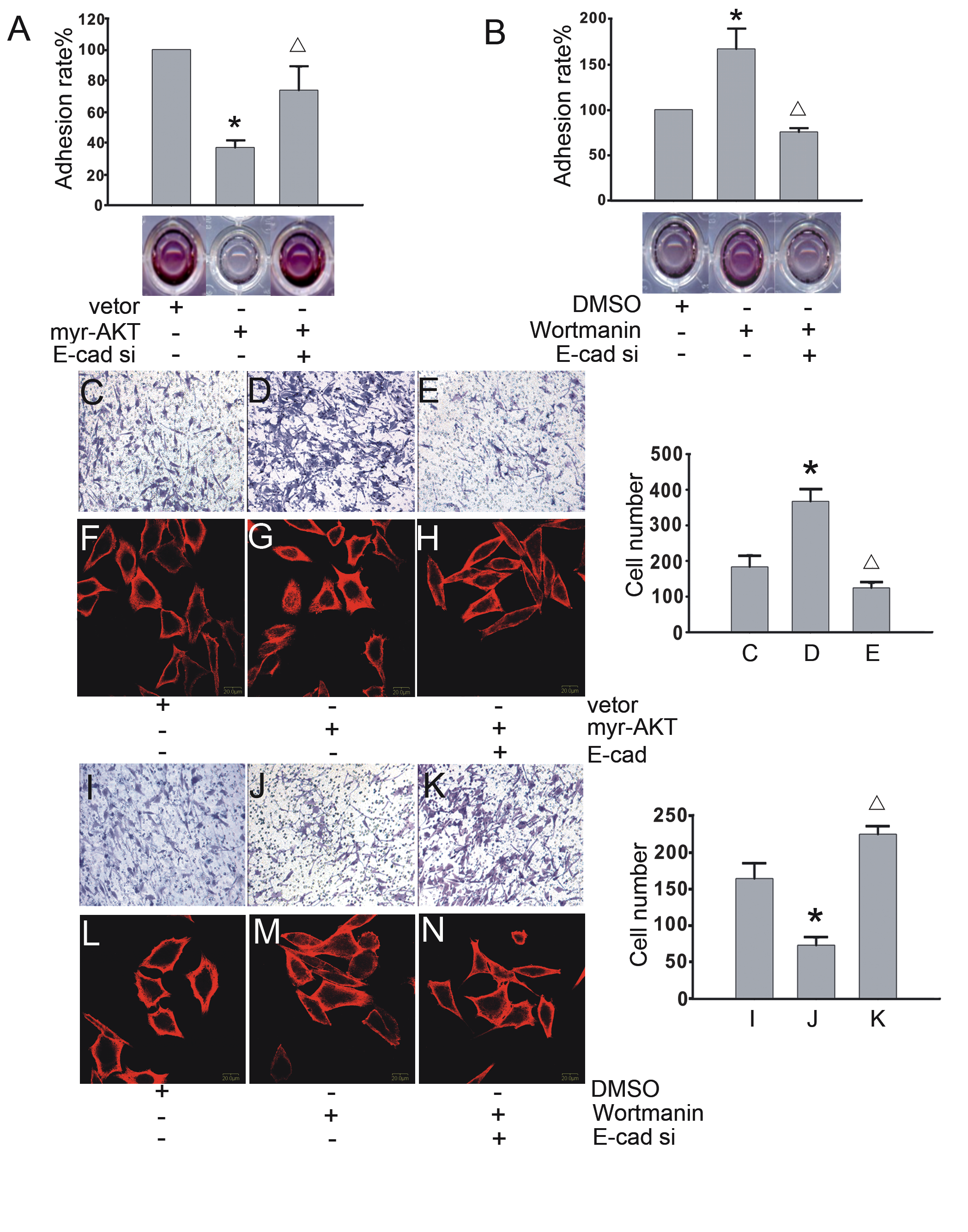

Supplement: S2 File — (TIF) [file pone.0266930.s002.tif]

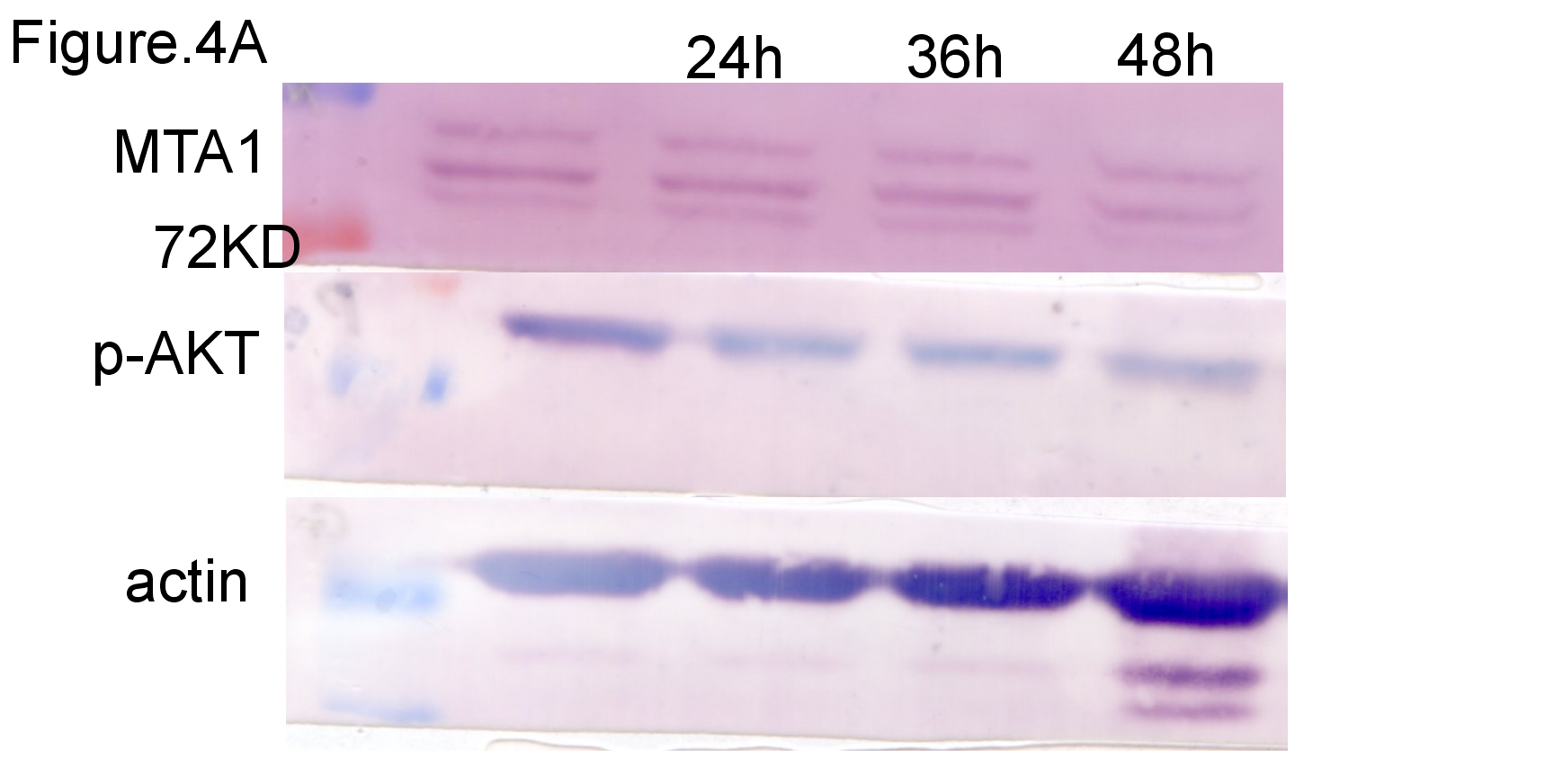

Supplement: S4 File — (TIF) [file pone.0266930.s004.tif]

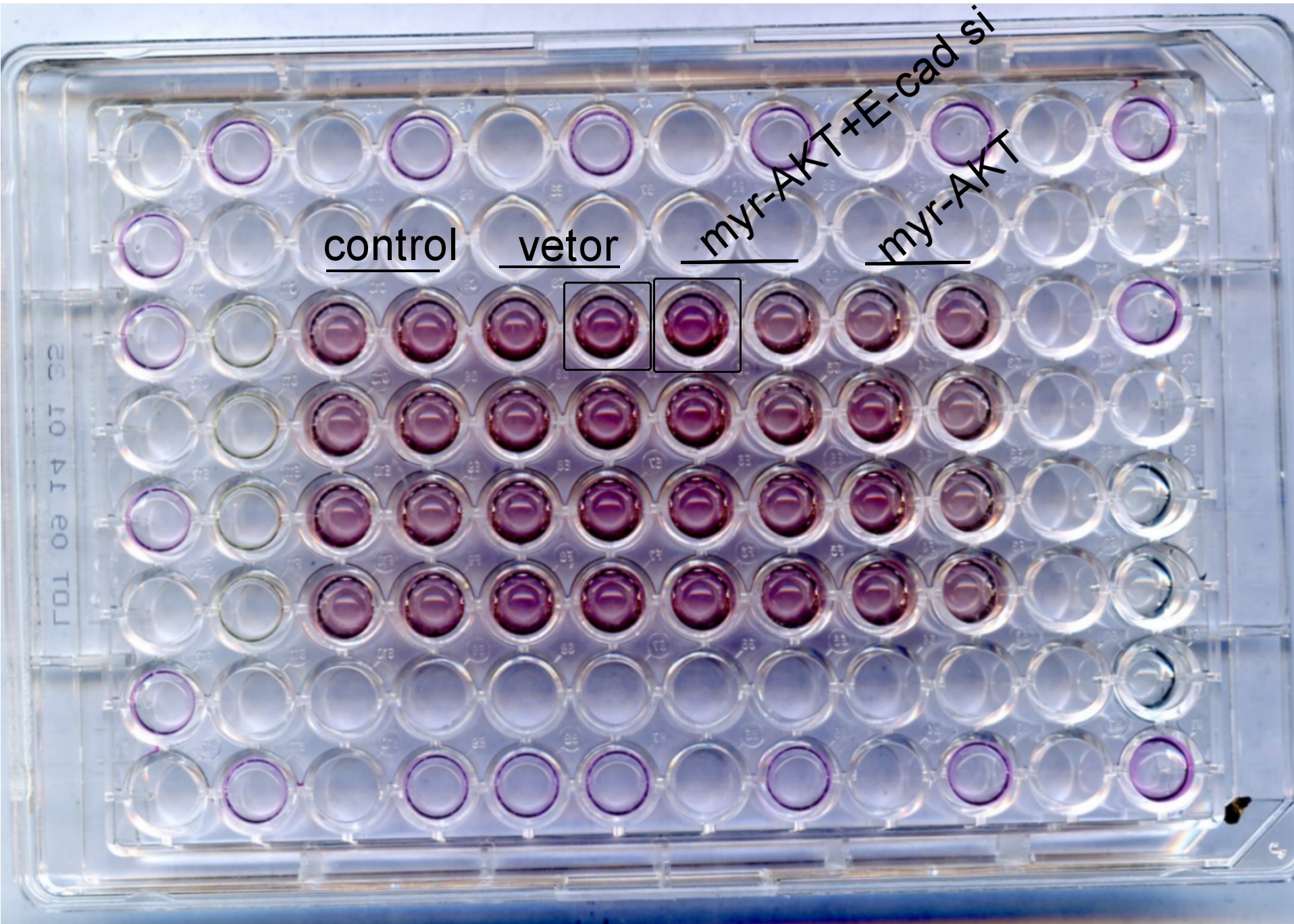

Supplement: S5 File — (JPG) [file pone.0266930.s005.jpg]
